# Molecular characterization of direct interactions between MPP1 and flotillins

**DOI:** 10.1038/s41598-021-93982-3

**Published:** 2021-07-20

**Authors:** Agnieszka Biernatowska, Paulina Olszewska, Krzysztof Grzymajło, Dominik Drabik, Sebastian Kraszewski, Aleksander F. Sikorski, Aleksander Czogalla

**Affiliations:** 1 Department of Cytobiochemistry, Faculty of Biotechnology, University of Wrocław, 50-383 Wrocław, Poland; 2 Department of Biochemistry and Molecular Biology, Faculty of Veterinary Medicine, Wrocław University of Environmental and Life Sciences, Norwida 25, 50-375 Wrocław, Poland; 3 Laboratory for the Biophysics of Macromolecular Aggregates, Department of Biomedical Engineering, Wroclaw University of Technology, 50-370 Wrocław, Poland; 4 Research and Development Center, Regional Specialist Hospital, Kamieńskiego 73a, 51-154 Wrocław, Poland

**Keywords:** Biochemistry, Biophysics, Cell biology, Computational biology and bioinformatics, Structural biology

## Abstract

Flotillins are the major structural proteins in erythroid raft domains. We have shown previously that the dynamic nanoscale organization of raft domains in erythroid cells may depend on flotillin-MPP1 interactions. Here, by using molecular dynamic simulations and a surface plasmon resonance-based approach we determined that high-affinity complexes of MPP1 and flotillins are formed via a so far unidentified region within the D5 domain of MPP1. Significantly, this particular “flotillin binding motif” is of key physiological importance, as overexpression of peptides containing this motif inhibited endogenous MPP1-flotillin interaction in erythroid precursor cells, thereby causing lateral disorganization of raft domains. This was reflected by both reduction in the plasma membrane order and markedly decreased activation of signal transduction via the raft-dependent insulin receptor pathway. Our data highlight new molecular details concerning the mechanism whereby MPP1 functionally links flotillins to exert their physiological role in raft domain formation.

## Introduction

Subcompartmentalization is a key feature of cellular membranes. Small and dynamic assemblies called membrane rafts form functional platforms involved in a wide array of cellular processes^1^. Their potential role in signaling and sorting is of particular interest in the context of cancer and new targets for anticancer therapies^2^. Membrane rafts differ in composition and biophysical properties from the bulk membrane as a result of preferential associations between sphingolipids (and/or saturated glycerophospholipids) and cholesterol. These are the driving forces for the formation of more ordered domains to which certain proteins and lipids are recruited^3,4^. Among a few peripheral proteins which are considered as markers of plasma membrane rafts are flotillins^5,6^. These proteins form multiprotein complexes at the cytosolic site of the plasma membrane comprising among others proteins of the MAGUK (membrane-associated guanylate kinases) superfamily, and thus contribute to raft domain assembly and dynamics.

On the other hand, widely expressed, peripheral scaffolding MAGUK proteins are specialized in organizing multi-protein complexes at the plasma membrane^7^. One of the essential features of MAGUKs is their ability to interact with proteins via highly conserved domains, arranged sequentially into a PDZ-SH3-GUK tandem. Such characteristic architecture allows them to act as molecular scaffolders, thereby enabling formation and clustering of numerous protein complexes at the cytosolic side of the plasma membrane that are crucial for maintaining the architecture of the plasma membrane or controlling specific signaling pathways^7,8^. MAGUK-based complexes have been implicated in numerous cellular processes such as maintaining cell polarity^9–11^, cell adhesion and intracellular signaling transduction^12,13^, synaptic plasticity and development^14–16^. Importantly, it has been shown that mutation in genes encoding MAGUKs or their target proteins are directly linked with numerous diseases, including cancer^17^, indicating therefore the importance of the MAGUK-driven contribution to the structural specialization of the plasma membrane and cell physiology.

**Figure 1:**
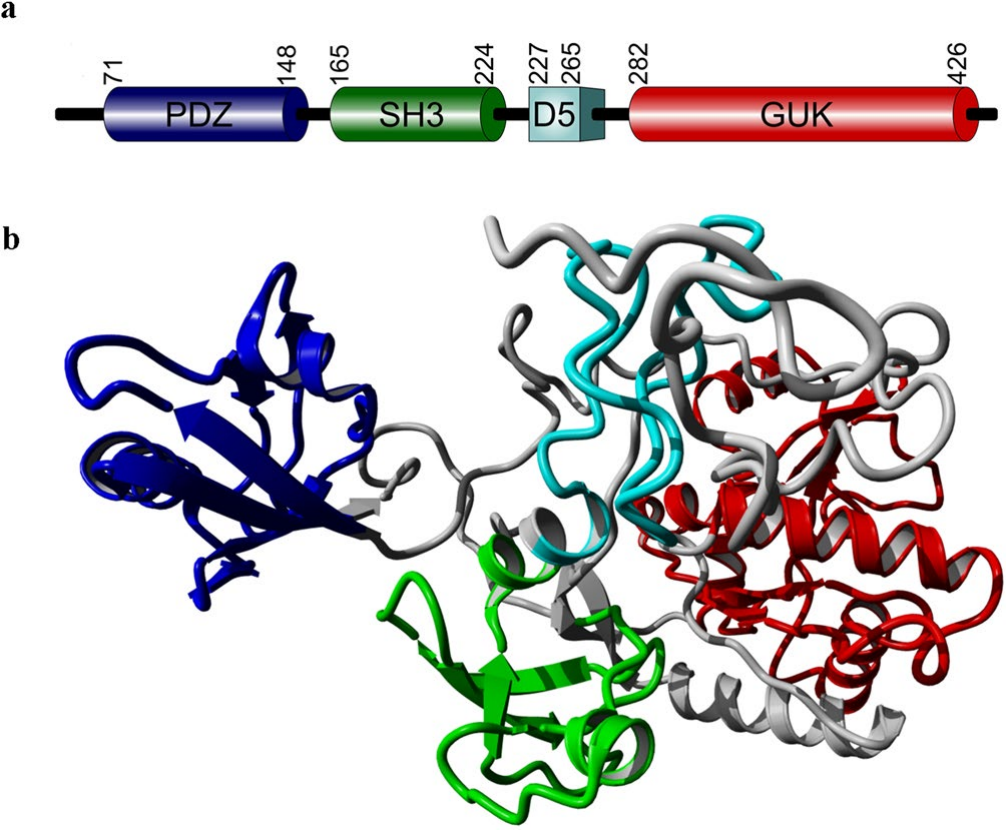
Structure of MPP1. Schematic representation (**a**) of the overall domain composition and ribbon model (**b**) of the structure of MPP1. PDZ blue, SH3 green, D5 light blue, GUK red. Domain boundaries were marked based on Quinn et al.^11^. Structural model redrawn according to Listowski et al.^44^.

Human erythroid MPP1(p55) belongs to the MAGUK MPP (membrane palmitoylated protein) subfamily^18^, and was originally identified in red blood cells (RBCs) as a major palmitoylated protein^19^. Sharing a characteristic, single PDZ-SH3-GUK module and additional D5 domain (Fig. 1), MPP1 was initially characterized as a key organizer of the junctional complex attaching a spectrin-actin-based skeleton to the RBC membrane lipid bilayer via integral proteins. In this case the N-terminal PDZ domain of MPP1 binds to the membrane protein, glycophorin C^20^, and the central D5 domain of MPP1 interacts with 4.1R protein^21,22^, thus forming a complex which is critical for maintaining the stability and mechanical properties of erythrocyte membrane. Significantly, our recent study performed on erythroid cells showed a novel physiological role of MPP1 in organizing functional raft domains. Using *MPP1* knockdown erythroid precursor HEL (human erythroleukemia) cells and giant plasma membrane vesicles (GPMVs) derived from them, we demonstrated that the marked decrease of MPP1 protein expression (or inhibition of its palmitoylation) is directly associated with significant changes in physicochemical properties of the plasma membrane monitored as an increase in membrane fluidity parameters and phase-separation properties^23,24^. This, in turn was correlated with noticeable loss in isolation of DRM (detergent resistance membranes) and marked reduction in activation of raft-dependent receptors and their downstream signaling pathways^25,26^. Detailed characterization of the molecular mechanism underlying the phenomenon of MPP1-dependent raft domain formation led us to identify the raft-marker proteins flotillin 1 and flotillin 2 as a new, direct MPP1-binding partners in RBC plasma membrane^23^. Importantly, these MPP1-flotillin interactions were shown to be physiologically relevant and independent from well-established, aforementioned interactions of MPP1 with 4.1 and glycophorin C, indicating a new role of the MPP1-flotillin linkage in stabilization of plasma membrane lateral heterogeneity in native RBC^23^. In fact, flotillins are important scaffolding components of the raft domains, playing a structural role in their organization^6,27^. The membrane-organizing capacity assigned to flotillins is due to their ability to form oligomers^28^ that serve as active assembly sites controlling numerous different cellular processes such as signaling^29–32^, endocytosis^33,34^, and cell adhesion^35^. These features emphasized flotillins as preferable molecular candidates for interacting with MPP1 in the context of plasma membrane lateral organization. Thus, based on these data, we proposed a concept where MPP1 and flotillins act as a driving force for functional raft domain formation in living cells. Our hypothesis assumes that MPP1 binds the pre-existing flotillins-nanoclusters/unstable rafts elements and therefore induces their fusion into larger nanodomains and stabilizes them as membrane rafts domains which become functional. Such rearrangement is connected to a change in membrane-lipid properties resembling formation of ordered domains and their separation from the bulk membrane. This hypothesis is in agreement with others^36^, however emphasize the major role of MPP1 in promoting oligomerization of flotillins, which in turn triggers co-assembly of flotillin-based oligomers and facilitates raft domain formation^23^. However, to build a comprehensive picture of this interesting novel mechanism, we decided to dissect the direct interaction between MPP1 and flotillins and precisely define the molecular details concerning their mutual binding capacity in vitro. Here, we demonstrated in vitro high affinity interactions between MPP1 and flotillin 1 or 2 and provided molecular details of this interaction by identifying a hitherto unknown “flotillin binding motif”’. Moreover, a recombinant protein corresponding to this domain via inhibiting this interaction reduced membrane order and markedly decreased activation of signal transduction via the raft-dependent insulin receptor pathway.

## Materials and methods

### Cloning, expression and purification of recombinant proteins

All the sequences of primers for the construction of plasmids used in this study are summarized in the Supplementary Table 1. The MPP1-truncated mutants (MPP1-Mut1-5) were subcloned into the pGEX-6p1 vector and expressed as soluble GST tagged proteins in *Escherichia coli* BL21 (DE3) or LEMO (DE) cells. Full length MPP1-GST and its truncated mutants were isolated in native conditions based on HBS buffers (10 mM HEPES, 150 mM NaCl, pH 7,4) and immobilized on glutathione-Sepharose 4B beads (GE Healthcare). The GST tag was cleaved off on the column with the PreScission protease (Sigma) according to the manufacturer's protocol. Purified recombinant proteins were validated by SDS-PAGE and Coomassie Blue staining (see Fig. S2). His-tagged flotillin 1 and 2 were purified under denaturation conditions as described previously^23^. After purification recombinant proteins were dialyzed into HBS-T (HBS-0.05% Tween-20) buffer and subsequently used for SPR binding study. For mammalian cell experiments the MPP1-Mut4-FLAG construct was additionally cloned into the p3XFLAG-CMV-10 vector (Sigma, St. Louis, MO).

### Real-time interaction analysis by surface plasmon resonance

**Figure 2:**
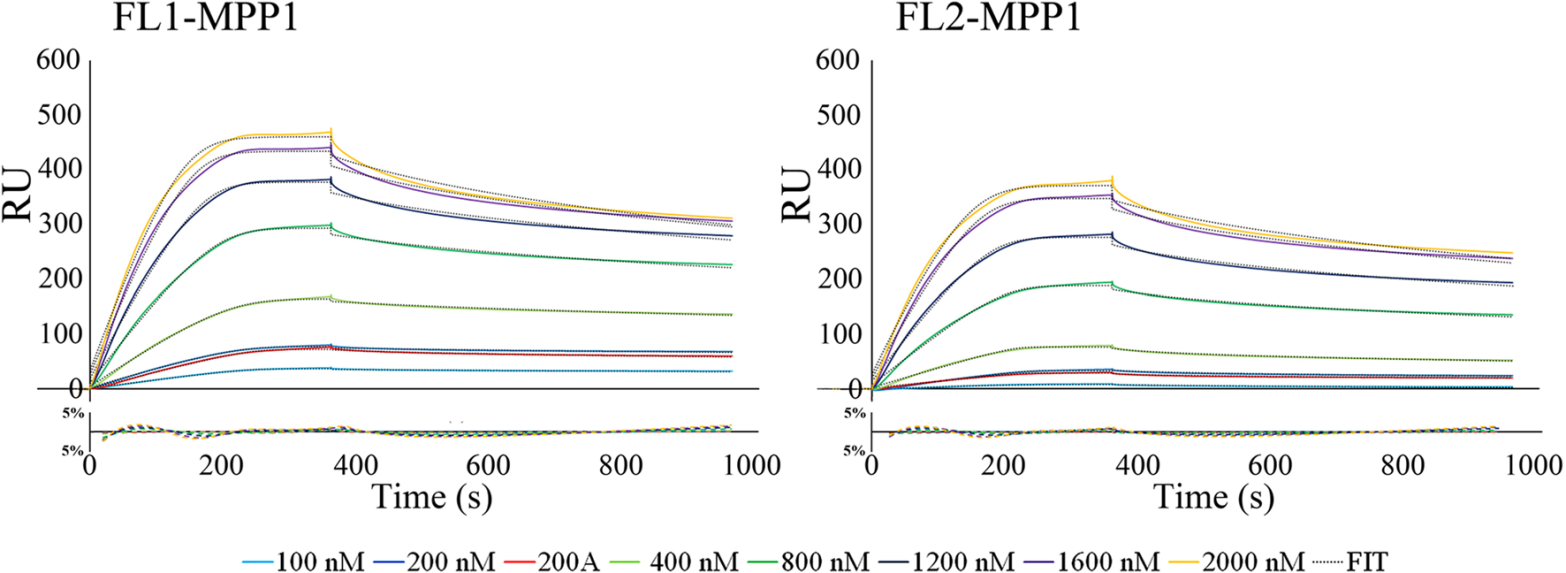
Recombinant MPP1 binds recombinant flotillin 1 or flotillin 2 with equilibrium dissociation constant, K_D_, values in nanomolar range. HIS-tagged flotillins were immobilized on the Ni–NTA chip and its interaction with full-length recombinant, untagged MPP1 (series of concentration 0–2000 nM) was analyzed by SPR using a BIAcore T200. Black dotted lines represent fitted 1:1 Langmuir binding model. Residual plots beneath every sensorgram series were evaluated for systematic divergences from the fitting curves. Other details in “Materials and methods” section.

The binding of full length MPP1 (Fig. 2) and its truncated mutants (Fig. 5) to recombinant His tagged flotillin 1 or flotillin 2 immobilized on Ni–NTA sensor chips (Series S sensor chip NTA; GE Healthcare) was analyzed by SPR using a BIAcore T200 (GE Healthcare) as described elsewhere^37^. Briefly, purified recombinant flotillin 1 or flotillin 2 was bound to the prepared surface via the His tag to a final level of approximately 4000 RU. To determine affinity of all analytes to flotillin 1 or flotillin 2, at least five different concentrations of each analyte (range from 50 to 2000 nM), as well as a sample buffer blank, were passed over the ligand-immobilized-(association phase—360 s) or the control empty chip surface followed by dissociation with running buffer (600 s). The resulting sensorgrams were obtained by subtracting the buffer blank from sample curves (recorded for the interactions of flotillins with MPP1 or its mutants) followed by substraction of sensorgrams obtained from empty chip surface. The equilibrium constants (K_D_) defined as a *k*_on_/*k*_off_ ratio were determined using BIAevaluation 3.1 software using global fitting and a 1:1 Langmuir binding model with an included mass transport step. Residuals were evaluated for systematic divergences from the fitting algorithms as a measure of the appropriateness of the binding model.

### Cell lines

HEL cells were kindly provided by Prof. M. Majka from Jagiellonian University of School Medicine. Cells were grown in RPMI 1640 medium supplemented with 10% fetal calf serum, 2 mM glutamine, 100 units/mL penicillin, and 100 μg/mL streptomycin at 37 °C in a humidified atmosphere of 5% CO_2_.

### Cell transfection and activation

Transient transfections of HEL cells were performed by CLB (Lonza, Basel, Switzerland) electroporation. Briefly, 2 × 10^6^ cells were transfected with empty p3XFLAG-CMV-10 vector (Sigma, St. Louis, MO) (FLAG-control) or MPP1-Mut4 plasmid respectively. 24 h post transfection cells were serum-starved for 20 h before treatment with human recombinant insulin (Gibco) [1 μg/ml] for 5 min, at 37 °C in a humidified atmosphere of 5% CO_2_. For immunoblotting, the stimulated cells were harvested, washed with ice-cold PBS and lysed for 30 min on ice in the lysis buffer (50 mM HEPES, pH 7.5, 100 mM NaCl, 1 mM EDTA, 10% glycerol, 1% NP-40) supplemented with 100 μM PMSF, protease inhibitor cocktail (Sigma-Aldrich) and phosphatase inhibitor cocktail (Santa Cruz). Proteins were separated by SDS-PAGE, followed by Western blot analysis. Immunodetection was performed using antibodies against phospho ERK (p-ERK 1/2), total ERK 1/2, and FLAG. Blots were quantified and normalized to appropriate loading controls using ImageJ software. All immunoblotting data presented here are representative of four independent experiments.

### FLIM analysis

FLIM was used to measure fluorescence lifetime values of a membrane-order sensitive probe, di-4-ANEPPDHQ (Life Technologies). After 48 h cells transfected with appropriate plasmids (FLAG transfected/control; MPP1-Mut4 transfected) were stained with 2 μM di-4 for 15 min at room temperature (RT), subsequently washed with HBSS buffer supplemented with 10 mM HEPES, pH 7.4 (Gibco) and transferred onto poly-l-lysine coated lab-TEK chambers and left for 20 min at RT. Such conditions resulted in labeling not only plasma membrane but also intracellular membranes, as shown in Fig. S8 and reported elsewhere^38,39^. FLIM measurements were performed at 23 °C using an LSM 510 META microscope (Carl Zeiss GmbH, Germany) upgraded with FLIM and FCS capabilities (PicoQuant GmbH, Germany) as described previously^23,25^. Briefly, 470 nm pulsed-laser with 40 MHz repetition rate was used for di-4 excitation and emission was detected with the use of long pass 505 nm emission filter. Average photon rate was approximately at the level of 10^4^–10^5^. Acquisition time was dependent on the intensity of the samples. Lifetime calculations of di-4 probe was measured only from the plasma membrane region (ROI; region of interest) as described previously^38,39^. Decay curves were fitted in the range up to 20 ns for all the experiments and the FLIM raw data were processed using SymPhoTime software (PicoQuant GmbH, Germany). Statistical analysis was performed using two-tailed unpaired t-test.

### CD analysis

Circular dichroism (CD) measurements were performed using a JASCO J-1500 spectrometer in a temperature-controlled cell (0.1 or 0.01 cm path length). All proteins were analyzed in HEPES-based buffer with addition of 0.1% Tween-20. Raw data were converted into molar ellipticity per residue using the equation: $$\theta =\frac{CD units }{10*n*p*c}$$, where CD units were in [mdeg] n-number of residues, p- path length in [cm], c- concentration in [$$\frac{\mathrm{mol}}{\mathrm{l}}$$].

### Flotillin 1 and flotillin 2 protein modelling

The sequences for flotillin 1 (O75955-1) and flotillin 2 (Q14254) proteins were obtained in FASTA format from UniProt. As no single complete protein structures of both flotillin 1 and 2 have been published, a model was built using I-TASSER software^40^. The obtained AA sequences of flotillin 1 and 2 were given as a query. The web server operated at its default setting, that is, no specification of templates was given. Also, no assignment of contact or distance restraints was given. The server was also set to not exclude any homologous or specific templates. Since the full structure of flotillin 1 remains unknown, the top template which was selected by the server was the SPFH domain of flotillin 2 (PDB: 1WIN). For flotillin 2 a protein from the liver vault was selected as a template (PDB: 4V60). To validate the correctness of the server selection, known fragments of flotillins (SPFH domains) were compared to those of 1WIN, 4FVF, 4FVJ, 4FVG and 3BK6 using the TM-align online tool^41^. They were found to be structurally similar (TM-score 0.72, 0.75, 0.88, 0.91 and 0.97 for flotillin 1; TM-score 0.95, 0.78, 0.82, 0.84, 0.64 for flotillin 2). Additionally, C-score and total energy were calculated. The top-ranked model constructed by the server was used in further in silico experiments.

### Molecular dynamics simulation

The full-atomistic molecular dynamics simulations were performed using NAMD 2.13^42^ software with CHARMM36 force field^43^ under NPT conditions (constant: number of particles, pressure, and temperature). Several systems were created for both stabilization of investigated proteins (flotillin 1 and flotillin 2) and investigation of interactions between them and the MPP1 protein model^44^. All systems were hydrated with TIP3P water molecules and ionized with 0.15 M NaCl. Stabilization of investigated systems was carried out for at least 100 ns for each of the investigated protein models. Simulations involving studying the interactions between the flotillin 1 and MPP1 proteins took, in total, 115 ns. For flotillin 2 they took 56 ns as the bindings occurred faster. Three dimensional periodic boundary conditions were applied in order to deal with potential energy disruption due to the origin cell discontinuity. More detailed descriptions of individual simulations are presented in Supplementary Information. To establish the binding between the flotillins and MPP1 a simple approach was used. For each simulation step possible binding sites were flagged if at least three MPP1/flotillin atoms from the amino acid group were within 3 Å of flotillin/MPP1 atoms.

## Results

### MPP1 binds flotillin 1 (FL1) and 2 (FL2) with high affinity in vitro

In our previous study we demonstrated that the newly identified linkage between MPP1 and flotillins contributes to the lateral plasma membrane organization in native RBCs^23^. Here, we decided to characterize the molecular details concerning MPP1-flotillin interaction and more precisely define their kinetic parameters and binding capacity in vitro using an SPR approach. Therefore, to examine the nature of individual interactions first, recombinant His-tagged flotillin 1 or flotillin 2 was immobilized on Ni–NTA biosensors and a series of concentrations of full length recombinant MPP1 was used as an analyte. The double-referenced sensorgrams were fit globally to a 1:1 kinetic binding model (Fig. 2), and calculated k_on_, k_off_ and K_D_ values, which represent the association rate, dissociation rate and equilibrium disassociation constant, respectively, are shown in Table 1.

**Table 1:** Kinetic parameters for the interaction of full length recombinant MPP1 or its truncated mutants with flotillin 1 and 2.

Interaction	Kinetic parameters		
	k_on_ (1/Ms)	k_off_ (1/s)	KD (M)
FL1-MPP1	4,50E+04	1,03E−03	2,28E−08
SD	3,80E+03	9,36E−05	5,48E−10
FL1-MPP1-Mut1	n/a	n/a	n/a
FL1-MPP1-Mut2	1,68E+03	1,18E−03	7,02E−07
SD	2,98E+02	2,29E−04	4,38E−08
FL1-MPP1-Mut3	3,75E+03	5,15E−04	1,37E−07
SD	5,73E+02	3,99E−05	3,46E−08
FL1-MPP1-Mut4	8,13E+03	3,19E−04	3,92E−08
SD	2,13E−02	3,04E−05	5,21E−09
FL1-MPP1-Mut5	n/a	n/a	n/a
FL2-MPP1	4,13E+04	1,28 E−03	3,10E−08
SD	8,64E+03	2,35 E−04	2,89E−09
FL2-MPP1-Mut1	n/a	n/a	n/a
FL2-MPP1-Mut2	1,63E+03	1,60E−03	9,82E−07
SD	4,04E+02	4,74E−04	8,51E−08
FL2-MPP1-Mut3	4,17E+03	8,28E−04	1,99E−07
SD	5,74E+02	1,52E−05	7,77E−09
FL2-MPP1-Mut4	5,60E+03	2,81E−04	5,02E−08
SD	9,71E+02	4,79E−05	7,30E−09
FL2-MPP1-Mut5	n/a	n/a	n/a

Obtained K_D_ values are averages of 3 independent series of experiments presented in Figs. 2 and 5 and were found to be independent of ligand concentration (see Figure S1).

Interestingly, the kinetic analysis revealed that both recombinant flotillin 1 and flotillin 2 interact with MPP1 with K_D_ in the nanomolar range where the interaction of MPP1 and flotillin 1 exhibited a K_D_ value of approximately 22.8 nM, while with flotillin 2 it showed a K_D_ of 31 nM (Fig. 2, Table 1). Significantly, the obtained K_D_ values were independent of analyte concentration, as presented in Fig. S1, and global calculations for K_D_ are similar to those calculated from individual curves for different analyte concentrations. Moreover, these particular data indicate a relatively high global association rate of binding MPP1 ~ 4.5 × 10^4^ 1/Ms for both proteins (see Table 1). We have to stress that the results of single concentration series were in good agreement with the data obtained by calculating the averages from all experiments for both flotillins. Thus, the results of the systematic approach presented here document the interaction parameters between MPP1 and flotillin 1 or flotillin 2 with high consistency, indicating that these proteins are able to form high-affinity complexes in vitro.

### Molecular dynamic (MD) simulations of MPP1-flotillin interactions

**Figure 3:**
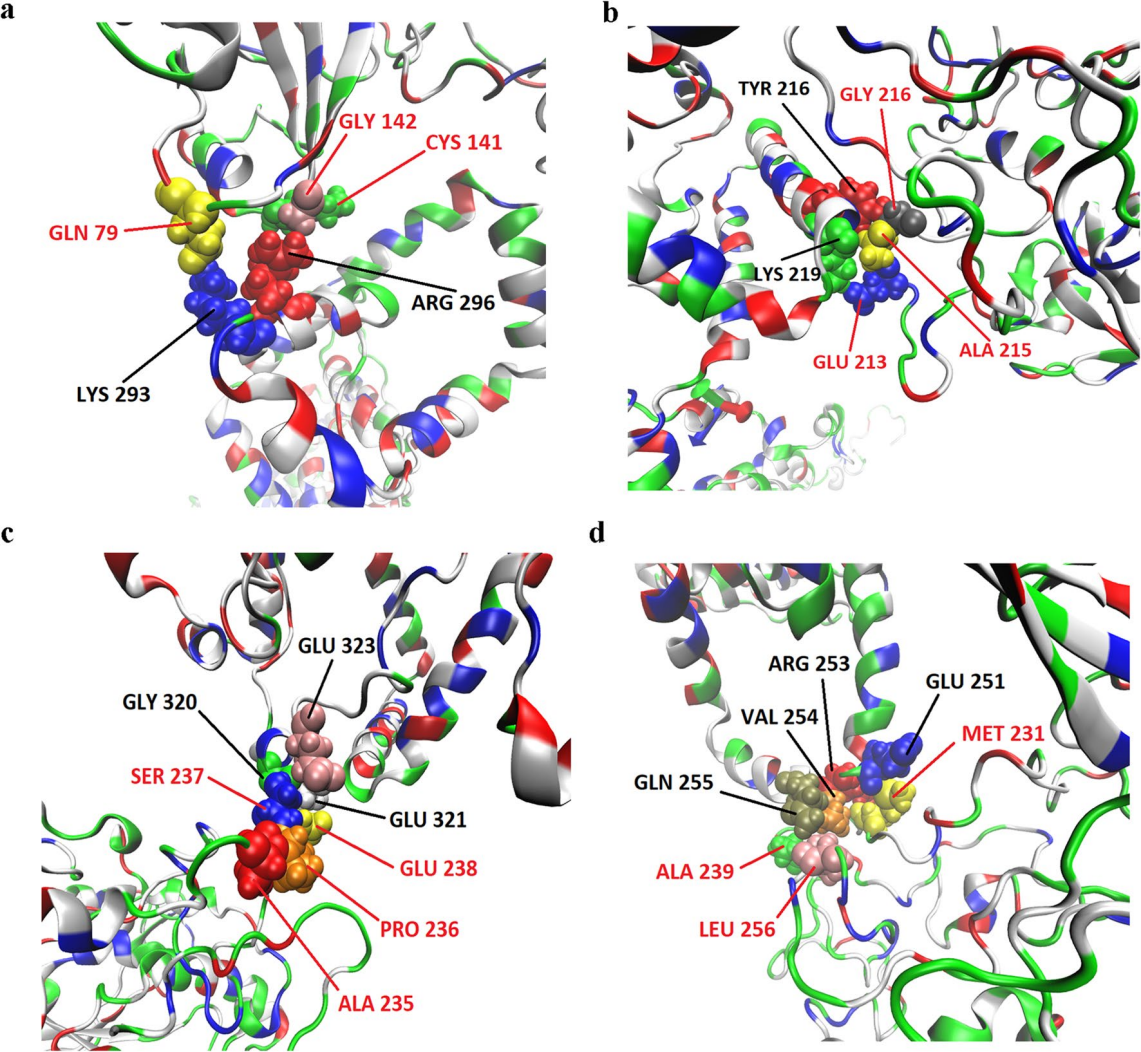
Visualization of MPP1-flotillin 1 binding sites mapped by molecular dynamics simulations. (**a**–**d**) Binding sites of MPP1 and FL1. MPP1 amino acid residues are marked in red, FL1 in black. Water molecules were removed for clarity.

**Figure 4:**
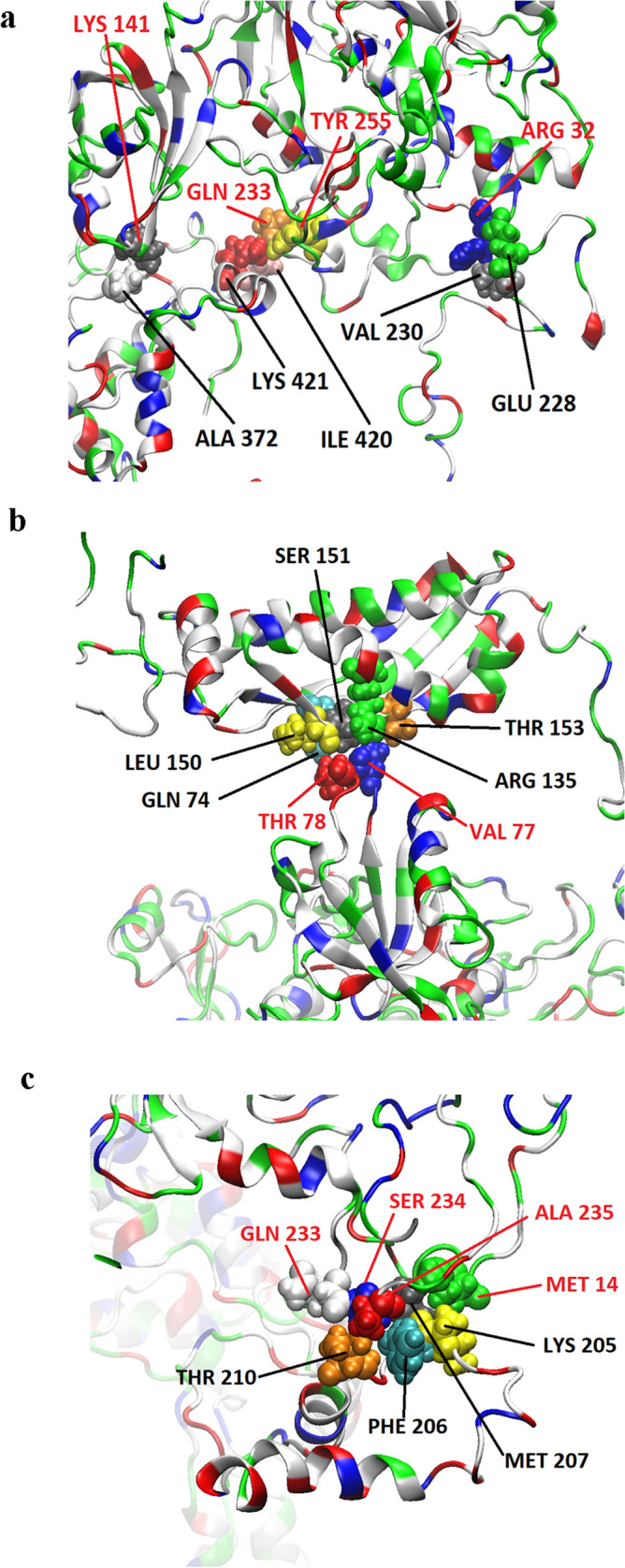
Visualization of MPP1-flotillin 2 binding sites mapped by molecular dynamics simulations. (**a**–**c**) Binding sites of MPP1 and FL2. MPP1 amino acid residues are marked in red, FL2 in black. Water molecules were removed for clarity.

In order to provide in-depth information about the potential binding site(s) involved in mutual MPP1-flotillin interactions MD simulations were carried out. For the purpose of these simulations both flotillin models were built from the sequence and tested (see Supplementary pdb files). Binding of the full length MPP1 protein^44^ to each of the flotillins was analyzed and several simulations were performed with different orientations in the simulation space (see Fig. S4 and S5). In the case of MPP1-flotillin 1 five potential binding sites were detected during experiments (Table S2); however, some of them exhibited relatively low electrostatic binding force strength (lower than ten percent of the highest values), and were considered unstable. The other, highly probable binding sites in molecular simulations involved Gln79/Cys141/Gly142 on MPP1 and Lys293/Arg296 on flotillin 1 (Fig. 3a), Glu213/Ala215/Gly216 on MPP1 and Tyr216/Lys219 on flotillin 1 (Fig. 3b), Ala235/Pro236/Ser237/Glu238 on MPP1 and Gly320/Glu321/Glu323 on flotillin 1 (Fig. 3c) and Met231/Ala239/Leu256 on MPP1 and Glu251/Arg253/Val254/Gln255 on flotillin 1 (Fig. 3d). In the case of MPP1-flotillin 2 simulations revealed four binding sites (Table S1) from which three were considered as stable binding sites; those were as follows: Arg32/Lys141/Gln233/Tyr255 on MPP1 and Asn228/Val230/Ala372/Ile420/Lys421 (Fig. 4a), Val77/Thr78 on MPP1 and Gln74/Thr135/Leu150/Ser151/Thr153 of flotillin 2 molecule (Fig. 4b) and Gln233/Ser234/Ala235/Met14 on MPP1 and Lys205/Phe206/Met207/Thr201 on flotillin 2 molecule (Fig. 4c).

### Mapping of a direct MPP1 interacting site for flotillins with SPR analysis

**Figure 5:**
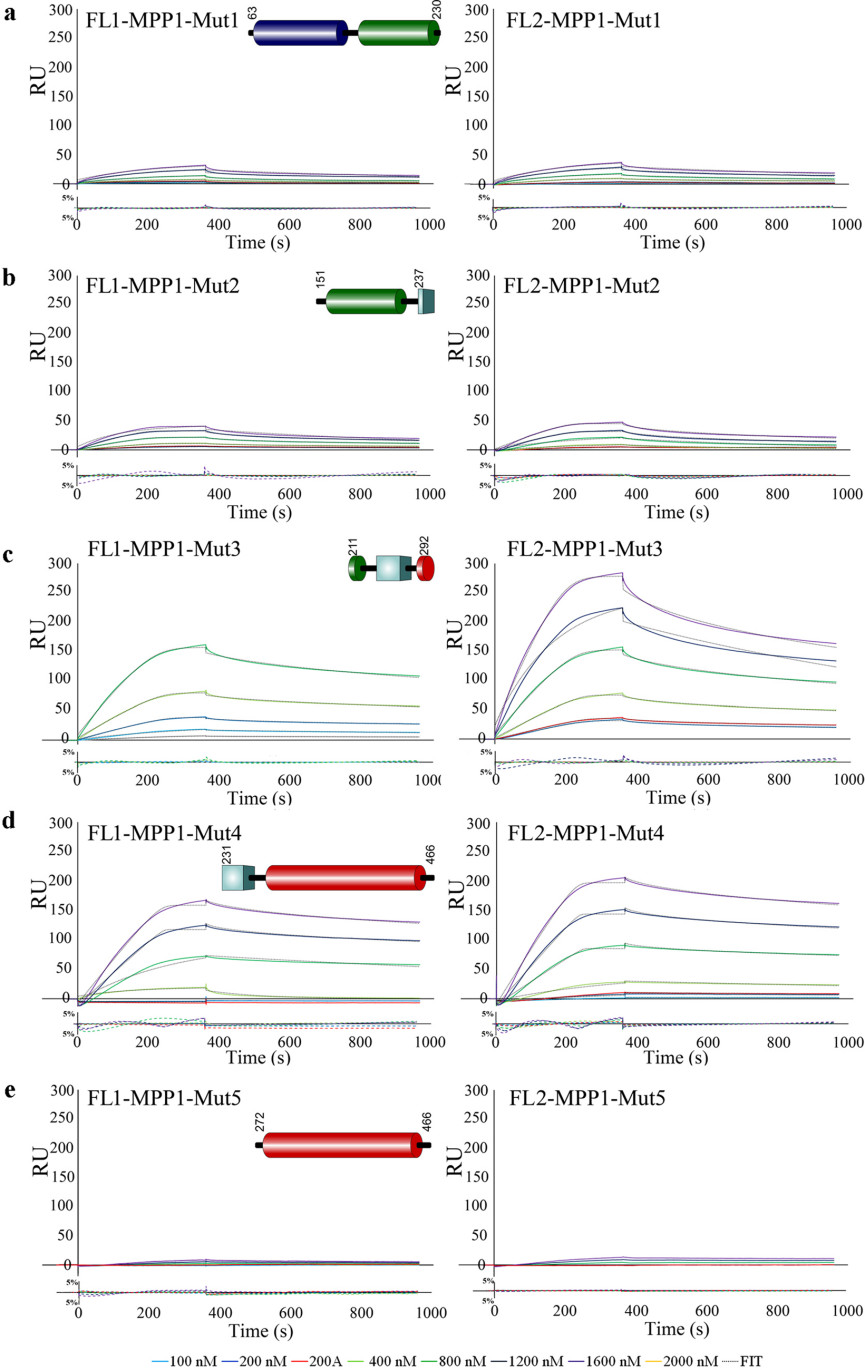
The D5 domain of MPP1 is of key importance for binding of flotillins. (**a**–**e**) HIS-tagged flotillins were immobilized on the Ni–NTA chip and their interactions with untagged MPP1 truncated mutants (MPP1-Mut1-5) in a series of concentrations 0–1600 nM were analyzed using a BIAcore T200. Other details as in Fig. 2 legend.

To evaluate the results obtained from our MD simulations, and check which of the above-mentioned potential binding sites might occur in vitro, we designed a series of MPP1-truncated mutants (Fig. 5), which were further used as analytes in SPR measurements to assess their individual binding with both flotillins. Purity of these recombinant proteins and their CD spectra are shown in Fig. S2 and S3. As shown in Table 1 and Fig. 5, from all analyzed MPP1 mutants, the highest affinity for flotillins was observed for MPP1-Mut4 (residues 231–466) (Fig. 5d). The calculated K_D_ value was 39.2 nM for flotillin 1, and 50.2 nM for flotillin 2 (Table 1). On the other hand, we also observed significant interaction between MPP1-Mut3 (residues 211–292) and MPP1-Mut2 (residues 151–237) with each of the flotillins. Here, the obtained K_D_ values of MPP1-Mut3 were approximately 137 nM for flotillin 1 and 199 nM for flotillin 2 (Fig. 5c, Table 1), while in the case of MPP1-Mut2 the binding was weaker, but still observable, and the K_D_ values were 702 nM and 686 nM, respectively (Fig. 5b, Table 1). The obtained K_D_ values were highly reproducible and in all cases independent of analyte concentration (Fig. S1). Importantly, our kinetic analysis of MPP1-Mut1 (residues 63–230) and MPP1-Mut5 (residues 272–466) virtually excluded the involvement of both the N-terminal and C-terminal regions of MPP1 in binding of flotillins, as no significant interactions were observed (Fig. 5a,e, Table 1). Notably, the sensorgram response curves revealed a very slow association even in high analyte concentrations, and therefore seemed to be the effect of non-specific binding in our experimental conditions rather than real interaction. Altogether, the above-presented kinetic parameters for MPP1 recombinant mutants highlighted that the central region of MPP1 is directly involved in flotillin 1 and 2 binding. Being more precisely based on the MPP1-mutant sequences and MD simulation data, we indicate the involvement of key amino acid residues in the MPP1 molecule, in particular in positions 231–238 and 256, which serve as critical flotillin binding sites. This particular region corresponds to the D5 domain of MPP1.

### MPP1-Mut4 affects plasma membrane order in living cells and modulates the raft-dependent signaling pathway

**Figure 6:**
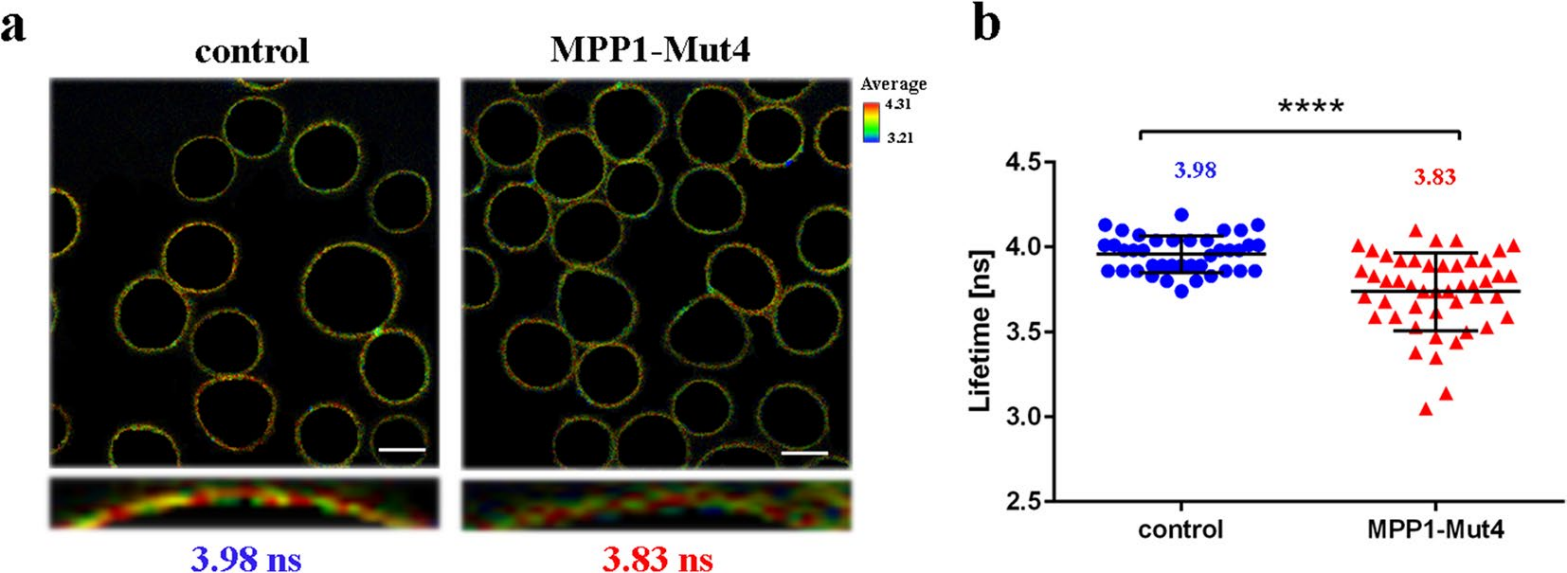
MPP1-Mut4 affects membrane order in HEL cells. Representative FLIM images (**a**) and quantitative analysis (**b**) of di-4 lifetime distribution collected from plasma membrane (ROI) of control (FLAG-transfected) and MPP1-Mut4-FLAG transfected HEL cells indicating significant (p < 0.0001) reduction in membrane order parameters after inhibition of endogenous MPP1-flotillin interactions. Each dot on a graph represents a single image of control (n = 38) and MPP1-Mut4-FLAG transfected cells (n = 42). Uncropped images, lifetime histograms as well as decay curves for representative images are shown in Fig. S8. Statistical analysis was performed using two-tailed unpaired t-test. Scale bar, 5 µm.

To assess the physiological significance of the newly identified “flotillin binding motif” we decided to test whether the MPP1-Mut4 has any effect on endogenous MPP1-flotillin interaction in living cells. Here, we took advantage of our well-described model system, the HEL cell line. Decreasing the level of MPP1 in these cells was previously shown to significantly affect raft domain formation, observed as a marked increase in plasma membrane and GPMVs fluidity parameters^24,25^. Therefore, to evaluate the possible biological impact of this fragment, HEL cells were transiently transfected with MPP1-Mut4-FLAG plasmid and control cells were transfected with “empty” FLAG plasmid. Subsequently, cells were labeled with the lipid bilayer order-sensing fluorescent dye di-4-ANEPPDHQ (di-4)^38^, the fluorescence lifetime of which is sensitive to membrane fluidity and analyzed in FLIM (fluorescence lifetime imaging microscopy). As shown in Fig. 6, FLIM data revealed statistically significant reduction in the lifetime value of the di-4 dye (~ 0.15 ns shift), in plasma membrane (ROI) of MPP1-Mut4 transfected cells (~ 3.83 ns) compared to the control (~ 3.98 ns). Such fluorescence lifetime shift of the di-4 probe is associated with an increase in membrane fluidity^38^. In this particular context it might be understood as partial disruption of MPP1-dependent raft domains due to the competitive binding of MPP1-Mut4 to endogenous flotillins, which eliminates the latter from interactions with endogenous MPP1.

**Figure 7:**
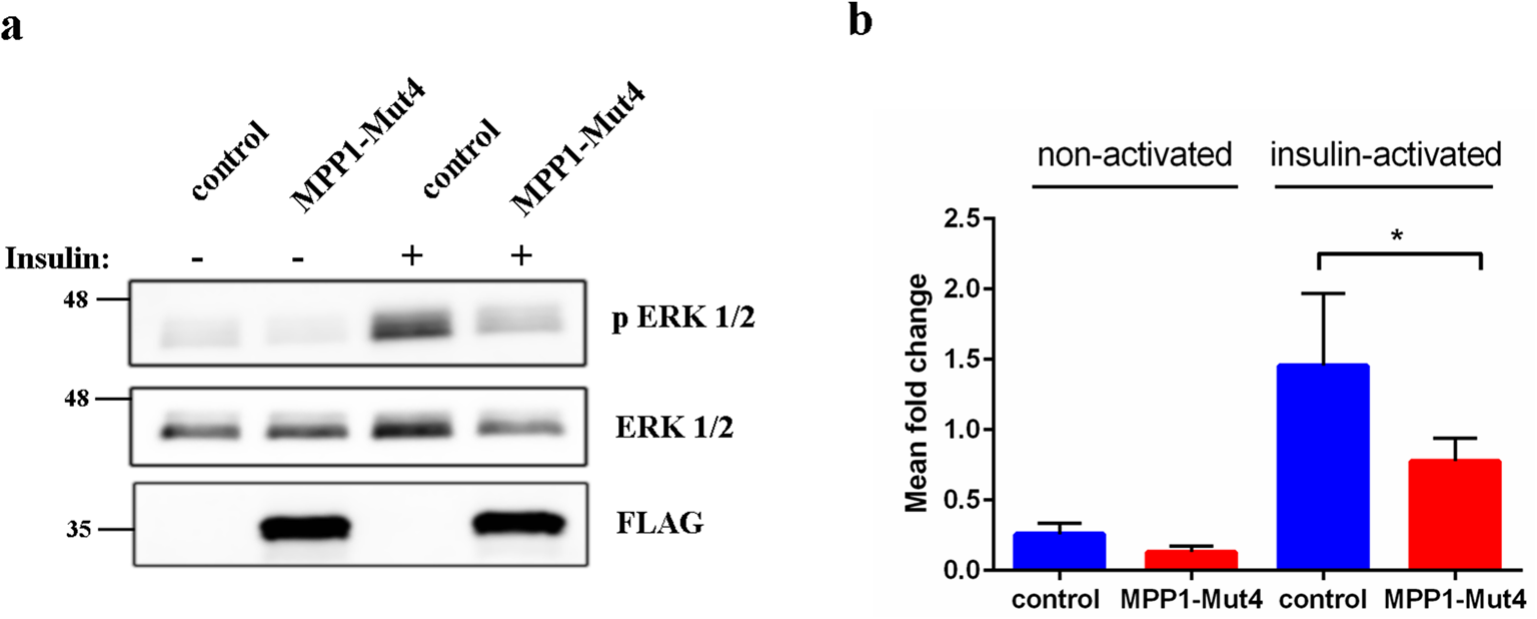
MPP1-Mut4 inhibits signal transduction from raft-dependent IR receptor in HEL cells. Control (FLAG-transfected) and MPP1-Mut4 transfected cells were treated with insulin and the whole-cell extracts were subjected to Western blot analysis and probed with antibodies against indicated proteins (**a**). Quantification of the relative phosphorylation levels of ERK1/2 in control and MPP1-Mut4 transfected cells (average ± S.D. from four independent experiments; statistical analysis was performed using non-parametric t-test p < 0.05) were performed (**b**). Uncropped blots are shown in Fig. S9.

As the disorganization of functional raft domains was reported previously to be correlated with impaired signal transduction from the activated raft-dependent insulin receptor (IR)^25,26^, to cross-check the “competitive” effect of MPP1-Mut4 on MPP1-flotillin interaction, the activation of IR with insulin was performed on transfected cells. As only the ERK1/2 signaling cascade downstream of the activated IR was shown to be MPP1-dependent in HEL cells^26^, the level of phosphorylated ERK1/2 (pERK1/2) was then monitored from whole-cell lysates after stimulation. Interestingly, we observed a marked reduction of pERK1/2 level in MPP1-Mut4 transfected cells (Fig. 7). The difference in activation level was approximately 46%, which may imply a partial competition of exogenously expressed MPP1-Mut4 and hence partial displacement of endogenous MPP1 from the flotillin-binding complex. Notably, these data are consistent with our previously described effect of *MPP1* knockdown on IR receptor activation and its downstream signaling in HEL cells^26^.

## Discussion

Our previous study showed that direct interaction between MPP1 and flotillins exists in the plasma membrane of RBC and functionally contributes to the organization of membrane rafts in living erythroid cells^23,25^. Namely, we showed that the decrease in functional MPP1 is correlated with di-4 lifetime in both plasma membranes of living cells and GPMVs derived from erythroid cells. Silencing of the MPP1 gene also led to a dramatic decrease in the DRM fraction^25^, but on the other hand, no changes in major lipid classes (including cholesterol and sphingomyelins) for plasma membrane-derived GPMVs could be observed^24^. Moreover, macroscopic phase separation in GPMVs obtained from MPP1-knockdown cells was observable approx. up to 15 °C, whereas control vesicles remained phase separated up to 17 °C^24^ confirming that MPP1 stabilizes more ordered phases and membrane phase coexistence. Next, flotillins, membrane raft marker proteins were found to be a direct binding partners for MPP1 in RBC plasma membrane^23^. These data opened a novel outlook in our understanding of the role of the MPP1 molecule as a critical regulatory and structural partner of raft-associated proteins^23^. The physiological relevance of formation of the MPP1-flotillin complexes in plasma membrane raised an important question about the molecular details underlying this phenomenon. Therefore, in this study we decided to dissect these interactions at the molecular level and precisely define the flotillins binding capacity of MPP1 and identify flotillin binding site(s) in vitro. Using a system of recombinant proteins and SPR-based kinetic analysis, we demonstrated for the first time the quantitative parameters of binding of recombinant MPP1 to both recombinant flotillin 1 and 2. Such an approach is particularly important, as so far no kinetic data have been available, although several flotillin-binding partners are known^5^. Interestingly, our data showed that the values of obtained equilibrium dissociation constants as well as the association and dissociation constants (k_on_ and k_off_) were similar in the case of both flotillins (Fig. 2, Table 1). This may be associated with a high degree of homology for flotillin 1 and 2, which share ~ 50% amino acid sequence identity^45,46^, which in turn might suggest that MPP1 probably binds homologous regions within flotillins (see below). It should be emphasized that the obtained K_D_ values were highly reproducible and in all cases independent of analyte concentration (Fig. S1), indicating that MPP1 and each of the flotillins form high-affinity complexes in vitro. Being interested which regions of MPP1 are directly involved in this binding, we performed the MD simulations of full length MPP1 and each flotillin. Structural models of the latter were built based on their sequences. This analysis revealed four, in the case of MPP1-flotillin 1 and three for MPP1-flotillin 2, presumable binding sites (Figs. 3, 4). Based on these data, a series of MPP1-truncated mutants were designed and their binding capacity towards each flotillin was further evaluated in SPR experiments. Such an approach enabled us to experimentally verify and more precisely characterize the “flotillin binding motif” within the MPP1 molecule. In particular, we found that three MPP1 mutants whose sequences share a common central region starting from residue 231 showed significant binding of flotillins in our SPR experiments. The highest affinity was observed for MPP1-Mut4 (231–466) (Fig. 5d, Table 1), and these values were close to those obtained for the full length recombinant MPP1 with each flotillin (Fig. 2). Over three times higher K_D_ values were observed for MPP1-Mut3, whose sequence included the range of residue 211–292 (Fig. 5c, Table 1). The weakest, although still detectable, interaction with flotillins was observed for MPP1-Mut2 (residues 151–237) (Fig. 5b, Table 1). This result is particularly interesting, as it suggests that the sequence of 7 amino acid residues between 231 and 237 have a considerable contribution to the affinity of MPP1 for flotillins (Fig. 5, Table 1). Notably, as no interaction with flotillins was detected for mutants comprising the N-terminus (up to residue 230) (MPP1-Mut1) or C-terminus (starting from residue 272) (MPP1-Mut5), we concluded that the “flotillin binding motif” is located between residues 231–271. Most precisely, together with MD simulation data we ascertained that amino acid residues 231, 235, 236, 237, 238 in the MPP1 molecule are engaged in stable binding with flotillin 1 (Fig. 3). In the case of flotillin 2 such binding is found for residues 233, 234, 235 and 256 in the MPP1 molecule (Fig. 4). Thus, we found that these key amino acid residues within the D5 domain of MPP1 are essential for high affinity interaction with flotillins. Moreover, further experiments with MPP1-Mut4 performed on the well-established erythroid HEL cell line (erythroblastoma) showed the biological relevance of this characteristic “flotillin binding motif”, which acts as an “endogenous competitor” for naturally occurring MPP1-flotillins complexes that can be observed primarily at plasma membrane of HEL cells, as determined in proximity ligation assay (PLA) (see Fig. S7 and supplementary movies). Such specific inhibition capability of MPP1-Mut4 was manifested as significant loss of membrane ordering parameters of the di-4 probe (~ 0.15 ns) compared to the control cells (Fig. 6). Importantly, these data are in line with our studies performed on RBC^23^ or HEL *MPP1*^24,25^. Furthermore, together with the changes in plasma membrane order, we also observed significant modulation of signal transduction via raft-dependent IR receptor signaling in cells transfected with MPP1-Mut4, where the level of the downstream activated pERK1/2 was approximately 50% lower compared to control cells (Fig. 7). Moreover, we also confirmed the inhibitory effect of recombinant MPP1-Mut4 on recombinant full-length MPP1-flotillin 1 interaction using competitive ELISA assay and bacterially expressed proteins (Fig. S6). Here, the inhibition effect of MPP1-Mut4 was approximately 15% when compared to MPP1-Mut1, and the magnitude of the competition effect was statistically significant. Taken together with the high affinity of MPP1-Mut4 for flotillins, it might imply that this mutant interferes with the endogenous MPP1 which forms complexes with flotillins and results in the disorganization of MPP1-dependent raft domains. Such competition leads to a significant decrease in plasma membrane ordering parameters and, as a consequence, affects raft-dependent signaling pathways.

The localization of such a newly mapped “flotillin binding motif”’ in MPP1 is particularly interesting, as so far the MPP1-D5 domain has been characterized as a main binding site for 4.1R protein in RBCs^21,22^, defining its primary role in maintaining the mechanical properties of RBCs. The high affinity of both 4.1R (70 nM^22^) and flotillins to the same region in the MPP1 molecule thus suggests its bifunctional involvement in different cellular processes. The question remains whether these molecules compete for binding to the D5 domain of MPP1 or rather such complexes can be formed independently of each other. Notably, our recent study indicated that the interaction of MPP1 with flotillins is independent of 4.1R binding^23^, which strongly suggests that MPP1 may simultaneously form two important types of complexes which independently control specialized functions within plasma membranes. Binding and stabilizing protein–protein complexes is a primary assigned function for the MPP subfamily. To fulfil this role, the conserved PDZ-SH3-GUK has been suggested to play key roles in multiple interactions with other molecules^13^. In erythroid cell membrane the role of the D5 domain is crucial. Of note, the D5 domain has been found in the structure of five (MPP1, MPP2, MPP5, MPP6, MPP7) out of seven members of this subfamily^18^. Such an additional domain might functionally distinguish these members from the others, allowing them to act as more versatile multifaceted organizers. However, when aligning amino acid sequences of the D5 domain of MPP members, particularly with respect to the putative “flotillin binding motifs”, we could not find any similarities. This, in turn, emphasizes that the “flotillin binding motif” in the D5 domain of MPP1 is unique. Therefore, the molecular characterization of direct interaction between MPP1 and flotillins brings us closer to defining key aspects of membrane organization and identifying novel potential therapeutic targets. This would be of particular interest in therapies of several tumors in which flotillin-dependent domains were shown to be closely associated with progression, development and metastasis^47^. Further systematic studies should consider experimental identification of the MPP1-binding site in flotillins; however, our MD simulation data indicate that C-terminal domains (flotillin domain) of each flotillin are involved in MPP1 binding (Figs. 3, 4). Given that the N-terminal region of flotillins mediates membrane binding and the C-terminal flotillin domain is responsible for the oligomerization of flotillins in living cells^5,6^, binding of MPP1 close to the C-terminal region could therefore mediate the latter process.

Although the high-affinity interactions between MPP1 and flotillins were observed in solution, the impact of lipid-bilayer cannot be excluded, since both proteins operate within or at the vicinity of plasma membrane of living cells (see Fig. S7). This fact is directly linked with the observed effects of the loss of MPP1 or MPP1-flotillins interactions in RBCs and HEL cells on the lateral organization of plasma membrane. Based on the model proposed by others^36^, and the fact that that functional raft domains are formed and stabilized temporarily upon internal factors (like oligomerization), we proposed a hypothesis which assumed the major role of MPP1 in oligomerization of flotillins assemblies into larger stable functional domains. Such specific MPP1-based sequestering/clustering process, may trigger the local changes in the organization of surrounding lipids, and attracting other molecules, resulting in physicochemical changes in plasma membrane^23,26^. It might be expected that the reported here high-affinity MPP1-flotillins interactions could be further strengthened at the cytoplasmic surface of plasma membrane due to multiple factors, including direct interactions of MPP1 and/or flotillins with lipids which may lead to reduced dimensionality. Indeed, results of some studies show that many protein–protein interactions can experience increases of effective affinities due to membrane localization^48^. This hypothesis should definitely point at further research directions in this field, which would require establishing new experimental models, such as MPP1 and/or flotillins reconstituted in proteoliposomes and immobilization-free technology to measure interactions.

To the best of our knowledge, this is the first report showing molecular details underlying formation of the MPP1-flotillin complex that opens a new outlook in our understanding of the involvement of MAGUK-scaffolding molecules and raft-marker proteins, flotillins, in the mechanism that governs the organization of functional raft domains. In fact, high affinity interaction of MPP1 to flotillins explains at least in part the biological ability of the formed complexes to maintain and modulate the properties of the plasma membrane, i.e. lateral membrane organization and its homeostasis. In other words, it becomes evident that these interactions are endogenous factors regulating raft domain formation in living cells.

## Supplementary Information

Supplementary Information 1.

Supplementary Information 2.

Supplementary Information 3.

Supplementary Video 1.

Supplementary Video 2.

Supplementary Video 3.

Supplementary Video 4.

Supplementary Video 5.

Supplementary Video 6.

## Data Availability

Further information and requests for resources and reagents should be directed to and will be fulfilled by the Lead Contact, Aleksander Czogalla (aleksander.czogalla@uwr.edu.pl). Materials and plasmids generated in this study are available upon request from the Lead Contact.
