# Supplementary material for: Molecular characterization of direct interactions between MPP1 and flotillins
Source: Sci Rep. 2021 Jul 20;11:14751. doi: 10.1038/s41598-021-93982-3 (PMC8292550; doi:10.1038/s41598-021-93982-3)
Supplement: Supplementary file 1 — Supplementary Information 1. [file 41598_2021_93982_MOESM1_ESM.pdf]

Molecular characterization of direct interactions between MPP1 and flotillins

Agnieszka Biernatowska [1,†] Paulina Olszewska [1,†], Krzysztof Grzymajło[2], Dominik Drabik [1], Sebastian Kraszewski [3], Aleksander F. Sikorski [4] and Aleksander Czogalla [1,*]

[1] Department of Cytobiochemistry, Faculty of Biotechnology, University of Wrocław, 50-383 Wroclaw Poland

[2] Wrocław University of Environmental and Life Sciences, Faculty of Veterinary Medicine, Department of Biochemistry and Molecular Biology, Norwida 25, 50-375 Wrocław, Poland

[3] Laboratory for the Biophysics of Macromolecular Aggregates, Department of Biomedical Engineering, Wroclaw University of Technology, 50-370 Wrocław, Poland

[4] Research and Development Center, Regional Specialist Hospital, Kamieńskiego 73a, 51-154 Wroclaw, Poland

***** Correspondence: aleksander.czogalla@uwr.edu.pl ; Tel.: +48 71 375 63 56

Supplemental Information

Table S1

Construct	Primer sequence	Plasmid
**MPP1-Mut1**	FP 5' TAGGATCCATGGGACAGGAGGTGCGGAAAGTGCGA 3'<br>RP 5' ATCTCGAGTTAACTTGCCACTCGCCATTCCTGCAG 3'	pGEX 6p1
**MPP1-Mut2**	FP 5' TAGGATCCATGAACCAGCAAAGCCGTCTTCCTGCACTACAG 3'<br>RP 5' ATCTCGAGTTAGCTAGGAGCTGACTGAGCCATACTTGCCACTCG 3'	pGEX 6p1
**MPP1-Mut3**	FP 5' TAGGATCCATGAATTGGTGGCAGGGACGGGTGGAAGGCTCC 3'<br>RP 5' ATCTCGAGTTACCCACTGGCTCCGATCAGCACCAGGGTCTT 3'	pGEX 6p1
**MPP1-Mut4**	FP 5' TAGGATCCATGGCTCAGTCAGCTCCTAGCGAAGC 3'<br>RP 5' ATCTCGAGTTAGGCTCCGATCAGCACCAGGGTCTTCCTCTT 3'	pGEX 6p1
**MPP1-Mut5**	FP 5' TAGGATCCATGGAGGAAGTCGTTCGGCTCCCTGCATTC 3'<br>RP 5'ATCTCGAGTTAGTAAACCCAGGAGACAGGCACCCACTG 3'	pGEX 6p1
**MPP1-Mut4-FLAG**	FP 5' ATGCGGCCGCAATGGCTCAGTCAGCTCCTAGCGAAGCCCCG 3'<br>RP 5' ATGGATCCTTAGTAAACCCAGGAGACAGGCACCCACTG 3'	p3xFLAG-CMV-10

Table S1. List of primer used to performed MPP1 mutants.

Table S2

Proteins subjected to MD simulations	Amino acid residues involved in binding		Binding strength [kcal/mol]
	MPP1	FL1	
MPP1-FL1	79/141/142	293/296	146 ± -19
	213/215/216	216/219	108.6 ± -5.5
	235/236/237/238	320/321/323	62 ± -13
	231/239/256	251/253/254/255	18 ± -3
	18/21/22	216/219/220	12 ± -4
MPP1-FL2	Amino acid residues involved in binding		Binding strength [kcal/mol]
	MPP1	FL2	
	32/141/233/255	228/230/372/420/421	84 ± -9
	77/78	74/135/150/151/153	12.4 ± -3.5
	233/234/235/14	205/206/207/210	11.2 ± -1.9
	123/127/233	366/415/416/418	4.8 ± -2.6

Table S2. Electrostatic binding force strength of MPP1-flotillin interactions determined using NAMD energy parser.

Figure S1

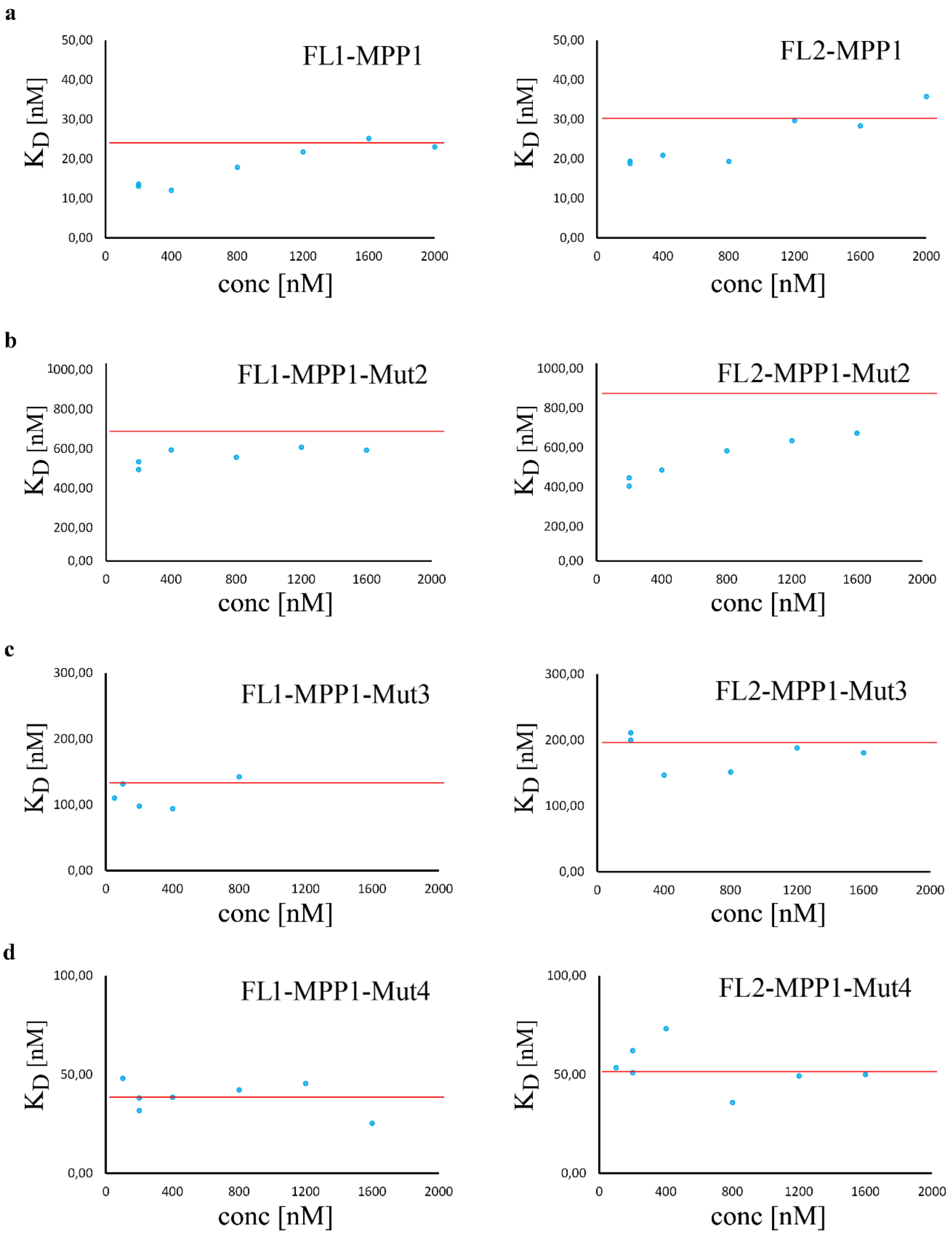

Figure S1. Plot of K_D values at different analyte concentrations [nM]

(A-E) K_D values marked as blue dots were obtained based on the 1:1 Langmuir binding model individual curves for each analyte concentration. Red lines represent the global K_D value.

Figure S2

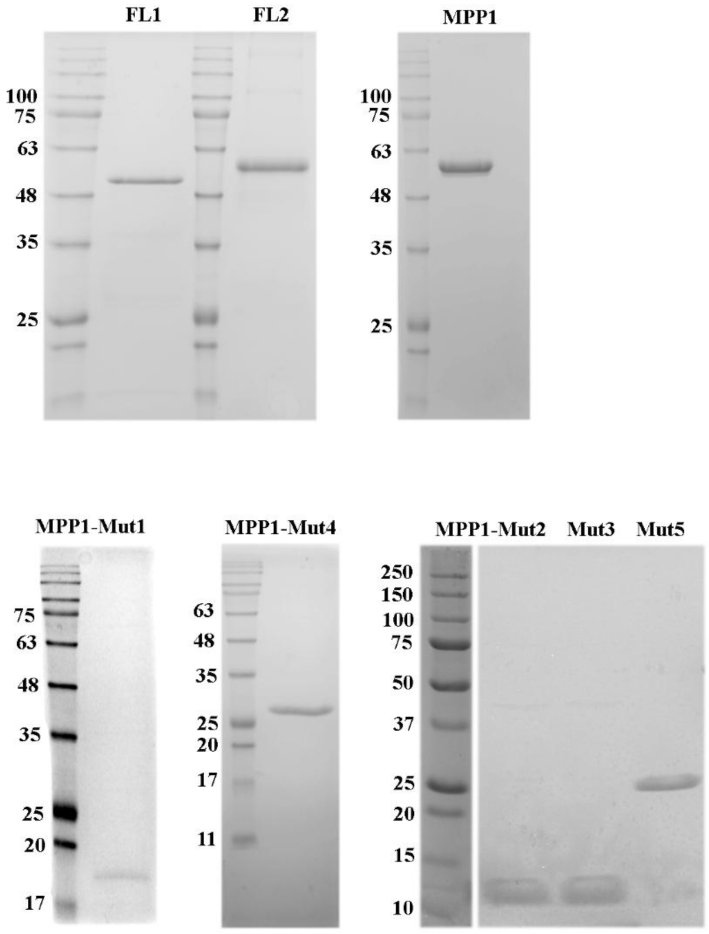

Figure S2. SDS-PAGE analysis of purified recombinant proteins used for SPR study

Coomassie blue staining of puriefied recombinant proteins used for SPR study and ELISA assay.

Figure S3

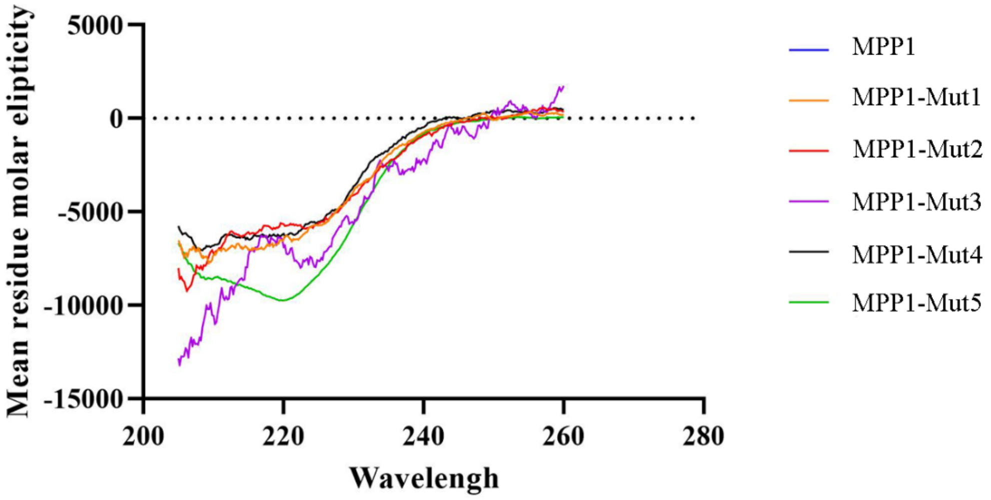

Figure S3. Circural dichroism of recombinant full length MPP1 and its truncate mutants

Circular dichroism far UV spectra of purified recombinant proteins, obtained at 20°C.

Figure S4

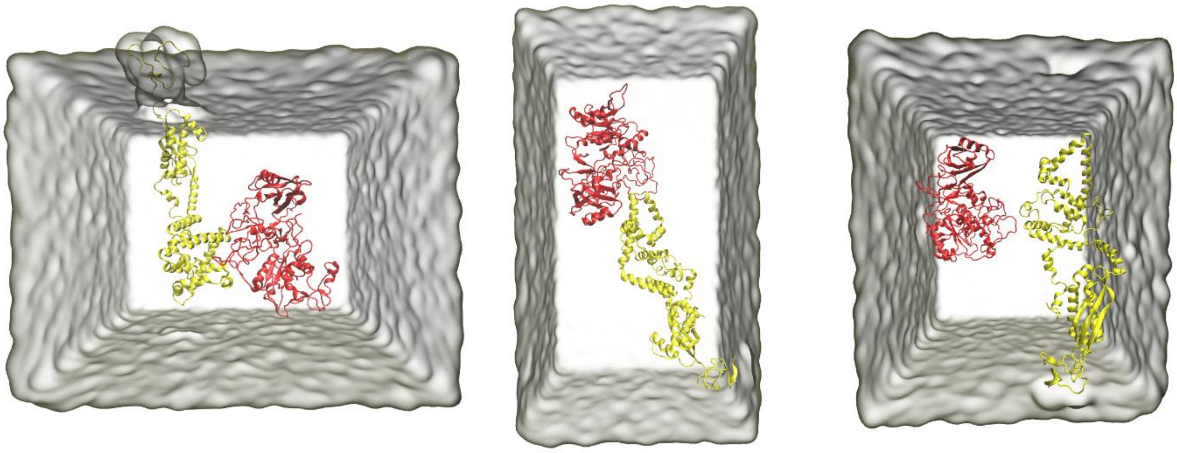

Figure S4. Final snapshots of selected molecular dynamics systems for studying the binding between flotillin 1 and MPP1 molecules. MPP1 molecule was marked in red, FL1 molecule was marked in yellow.

Figure S5

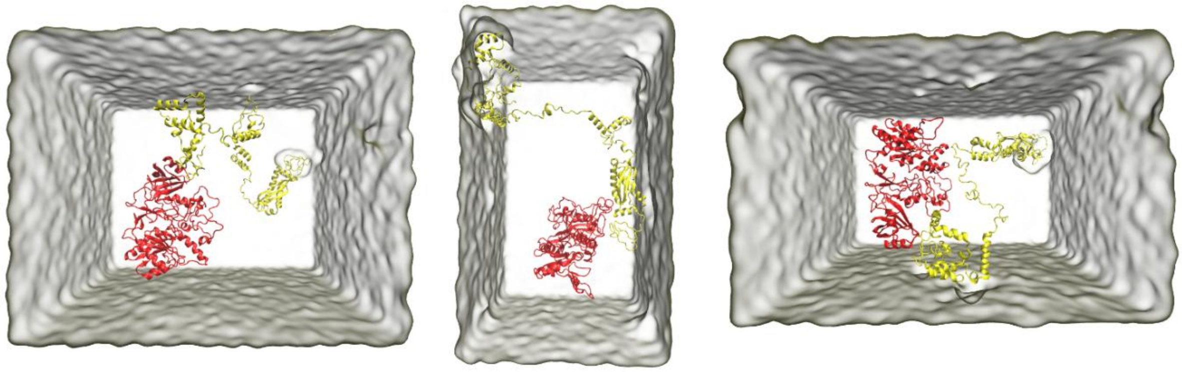

Figure S5. Final snapshots of selected molecular dynamics systems for studying the binding between flotillin2 and MPP1 molecules. MPP1 molecule was marked in red, FL2 molecule was marked in yellow.

Figure S6

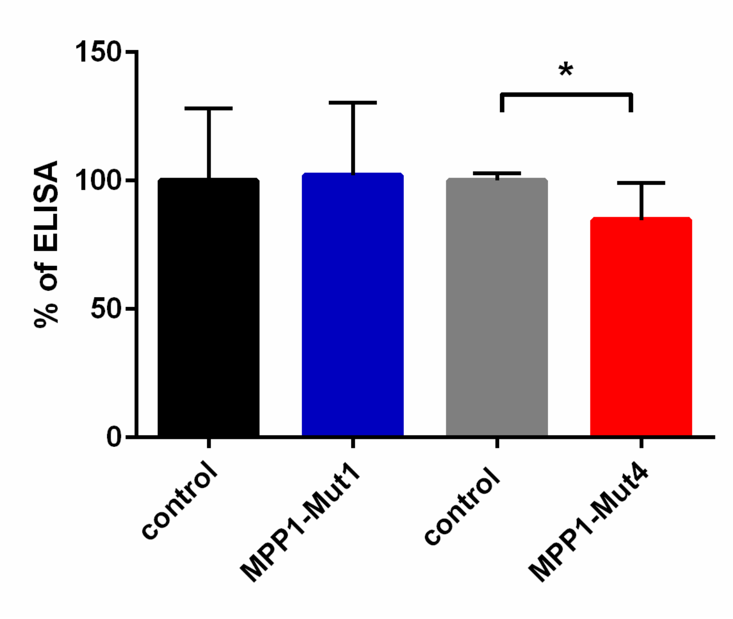

Figure S6. Inhibitory effect of MPP1-Mut4 on recombinat MPP1-flotillin 1 interaction using ELISA assay

Competition ELISA assay show inhibitory effect of MPP1-Mut4 on binding the full length recombinant MPP1 to FL1. Briefly, ELISA assay was performed in 96-well plates. Recombinant FL1 (2 µg) was non-specifically adsorbed on a plate using carbonate buffer (3 mM Na_2CO_3, 7 mM $NaHCO_3$, pH 9,6) for 1 h, RT. Then, the plate was washed 3 x 5 min with HBS-T and blocked overnight with 2% BSA at 4°C. After washing 3 x 5 min with HBS-T the mixture of constant concentration of recombinant full length MPP1 (100 nM) and MPP1-Mut4/MPP1-Mut1 ratio (1:50) was applied and incubated for 1 h at room temperature. Binding of MPP1 to FL1 without "competitor" was used as a positive control. Plate was washed 3 x 5 min with HBS-T followed by incubation with mouse anti-MPP1 antibodies (Abnova, 1:2000, 1h, RT) and rabbit anti-mouse HRP-conjugated antibodies (Jackson Antibodies, 1:10000, 30 min, RT). TMB kit (Thermo Fisher Scientific) was used for visualization. Reaction was performed for 15 min and stopped using 2 M sulfuric acid. Reading was performed on Rayto Plate reader at 450 nm. Statistical analysis was performed using non-parametric Student's t-test ($p < 0.05$).

Figure S7

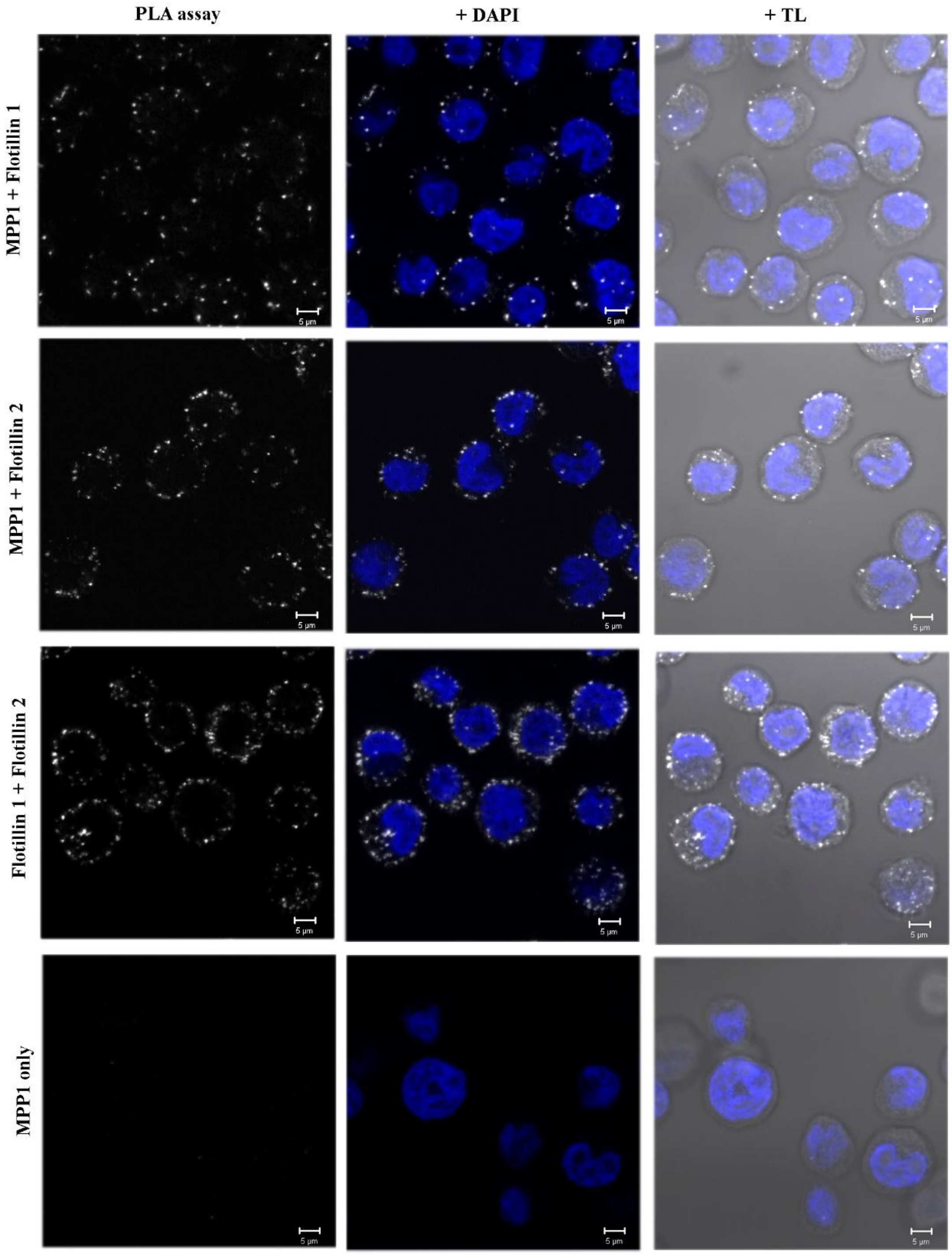
PLA assay
+ DAPI
+ TL
MPP1 + Flotillin 1
MPP1 + Flotillin 2
Flotillin 1 + Flotillin 2
MPP1 only
5 μm

Figure S7. Visualization of direct close-proximity interactions between MPP1 and flotillins in HEL cells performed by the PLA assay.

Briefly, HEL cells were fixed on poly-L-lysine coated cover-slips then permabilized with 0.1% Triton X-100 solution and subsequently blocked with Blocking solution (Sigma) for 30 min at RT. Cells were then probed with the two selected primary antibody pairs: anti-MPP1(Abnova, CAT#H00004354- M01)/flotillin 1 (Abcam, CAT#ab41927) or anti-MPP1/flotillin 2 (Abcam, CAT#ab96507), respectively, at 4 °C overnight in a humid chamber. After incubation cover-slips were washed and processed with the Duolink-PLA proximity ligation assay (Sigma) according to the manufacturer's protocol. As a positive control, HEL cells were incubated with anti-flotillin1 and anti-flotillin2 (Abcam, CAT#ab20399) antibodies and PLA probes, whereas as a negative control, fixed cells were stained with one primary antibody followed by PLA probe incubation (anti-MPP1; MPP1 only) according to the manufacturer's protocol. Endogenous close-proximity complexes between proteins are shown as gray dots, nuclei were stained with DAPI (producent???). Imaging was performed with a LSM 510 META confocal microscope (Carl Zeiss, GmbH Germany) using a PLAN-APOCHROMAT 63x/1.4 OIL DICM27 objective. All images were contrast-enhanced using ZEN 2009 Light Edition software. Images represents the central z-stack position. TL – transmitted light. Scale bar, 5 μm. Z-series movies are attached as separate supplementary files.

Figure S8

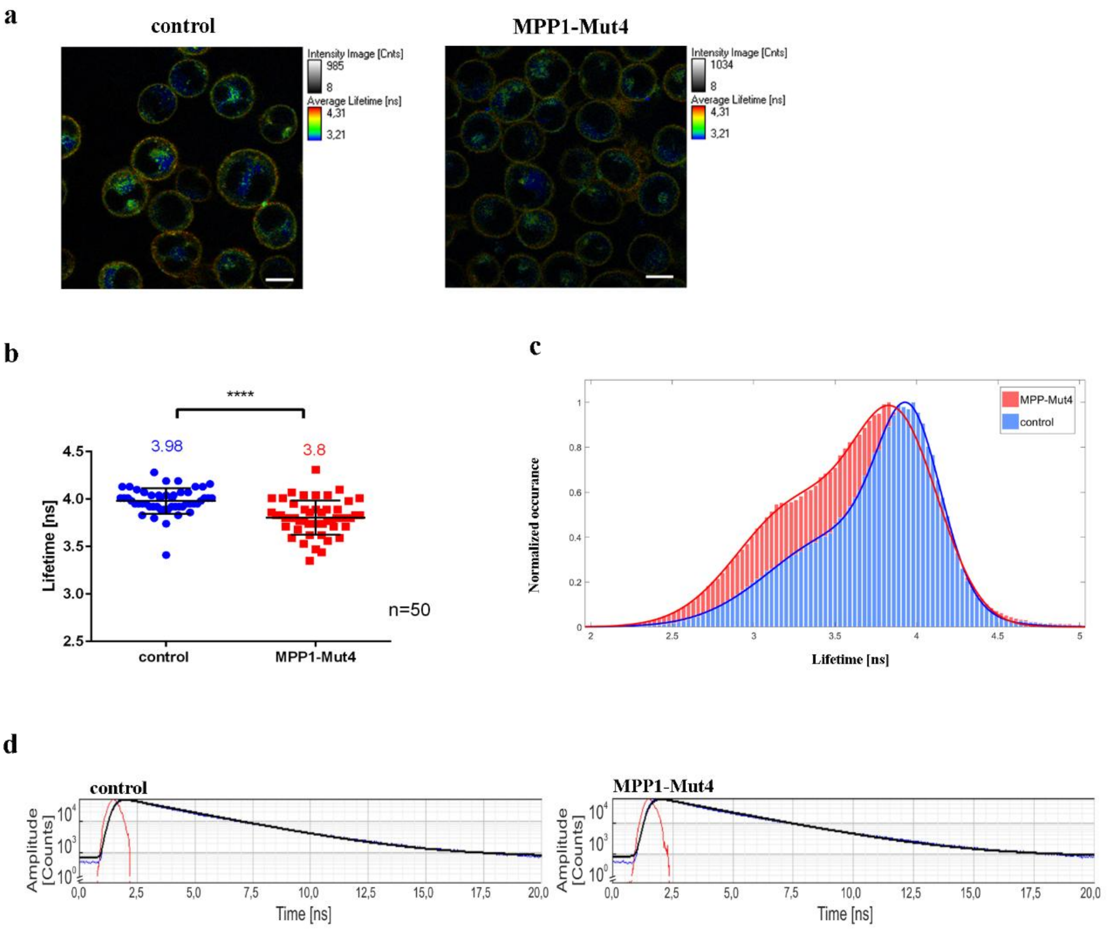

Figure S8. Supplementary data for FLIM experiments

a) Original/uncropped FLIM images of di-4-stained control and MPP1-Mut4 transfected HEL cells presented in main manuscript Figure 6. Scale bar, 5 µm. **b**) Individual data points and mean SD representing di-4 lifetime value of 50 randomly selected control cells (ROI) or MPP1-Mut4 transfected cells (ROI). p<0.0001 computed from unpaired, two-tailed test. **c**) Lifetime histograms for di-4-stained HEL control and MPP1-Mut4 transfected cells presented in main manuscript Figure 6. Fitting was performed by two-terms Gaussian using Levenberg-Marquardt algorithm. **d**) Lifetime decay curves and the one-component regression fitting (single exponential) of di-4-stained HEL control and MPP1-Mut4 transfected cells (plasma membranes) presented in Figure 6 (blue – TCSPC histogram, red – instrument response function (IRF), black – fitted curve).

Figure S9

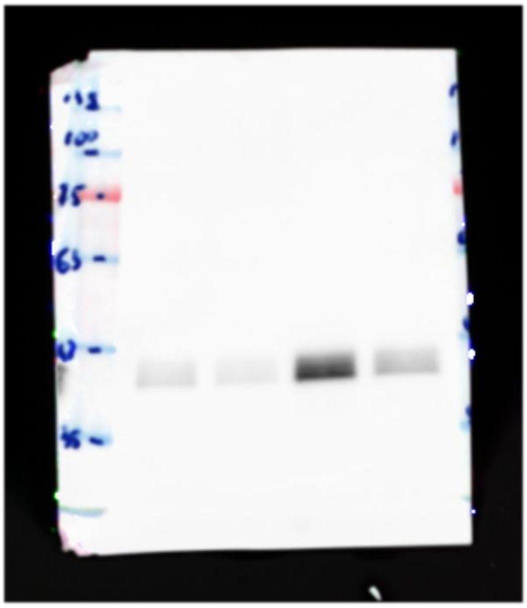

p ERK 1/2

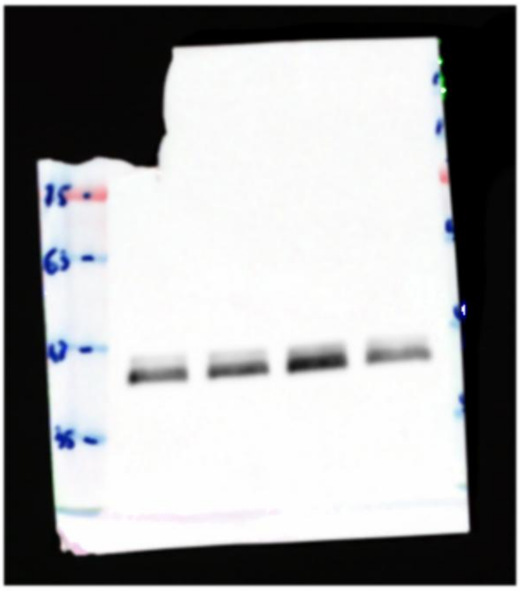

ERK 1/2

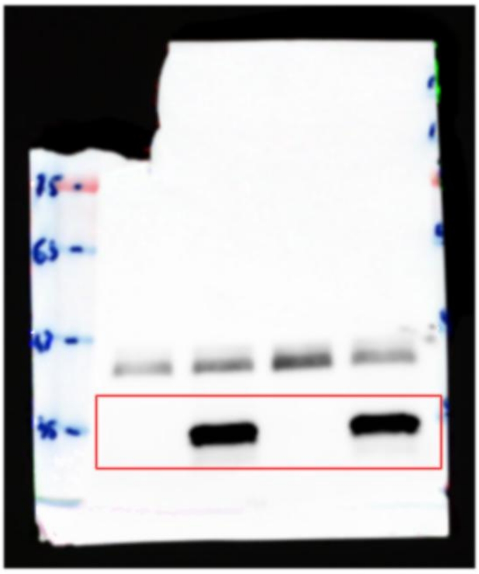

FLAG

Figure S9. Full-length blots form Figure 7.

Antibodies used: Mouse monoclonal anti p44/42 MAPK (ERK1/2) Cell Signaling Technology CAT#4696S; Rabbit monoclonal anti phospho-p44/42 MAPK (ERK1/2) Cell Signaling Technology CAT#4370S; Mouse monoclonal anti FLAG (M2) Sigma-Aldrich CAT#F1804; Membrane stained with anti ERK ½ antibodies was stripped and re-blotted with anti FLAG antibody (red frame).
